# Sex and age differences of inflammatory biomarkers around a bloodstream infection: a population-based cohort study

**DOI:** 10.1007/s15010-026-02732-y

**Published:** 2026-01-20

**Authors:** Cathrine Sandager Budtz, Line Riis Jølving, Pedro Póvoa, Stig Lønberg Nielsen, Ram Benny Dessau, Jens Kjølseth Møller, John Eugenio Coia, Kim Oren Gradel

**Affiliations:** 1 Center for Clinical Epidemiology, Odense University Hospital, Kløvervænget 30, Entrance 216, Ground Floor, 5000 Odense C, Denmark; 2 Research Unit of Clinical Epidemiology, Department of Clinical Research, University of Southern Denmark, Kløvervænget 30, Entrance 216, Ground Floor, 5000 Odense C, Denmark; 3 The Polyvalent Intensive Care Unit, Hospital de São Francisco Xavier, CHLO, Estrada do Forte do Alto do Duque, Lisbon, Portugal; 4 NOVA Medical School, New University of Lisbon, Lisbon, Portugal; 5 Department of Infectious Diseases, Odense University Hospital, Odense, Denmark; 6 Research Unit of Infectious Diseases, Department of Clinical Research, University of Southern Denmark, Odense, Denmark; 7 Department of Clinical Microbiology, Zealand University Hospital, 4600 Køge, Denmark; 8 Department of Clinical Microbiology, Vejle Hospital, University Hospital of Southern Denmark, Vejle, Denmark; 9 Institute for Regional Health Research, University of Southern Denmark, Odense, Denmark; 10 Department of Radiology, University Hospital of Southern Denmark, Kolding, Denmark

**Keywords:** Sex differences, Age differences, CRP trajectories, Neutrophil trajectories, BSI, Postmenopausal

## Abstract

**Purpose:**

Few studies in humans have revealed differences in the inflammatory responses between biological sexes when encountering serious infections. Our study aimed to investigate how those differences were presented among sexes and age groups from 30 days before (D–30) through 30 days after (D30) a bloodstream infection (BSI).

**Methods:**

We did a retrospective population-based cohort study, including patients aged > 15 years with their first-time BSI between 2007 and 2016.

Based on aggregated data, we computed daily mean levels of C-reactive protein (CRP) and neutrophils in the D–30/D30 period, separately for females and males within the age groups 15–49 and ≥ 50 years. For each age group, we used adjusted multilevel mixed effects linear regression analyses to detect differences in daily mean levels between females and males.

**Results:**

A total of 24,074 patients had 268,648 specimens with CRP and 138,482 with neutrophils. CRP and neutrophils peak values were significantly higher in females, reaching their highest values among the ≥ 50 years group. For all age groups, peak values occurred for CRP at D1 and for neutrophils at D0. Neutrophil values were more equal between the sexes, although higher levels were found in the ≥ 50 year age group among females after D–4.

**Conclusion:**

Females and males with BSI exhibited different trajectories and different peak values close to the BSI episode, in particular in females in the ≥ 50-year age group. Severe infections, such as BSI, need further investigation regarding sex differences, stratified into age groups for expected female menopause.

**Supplementary Information:**

The online version contains supplementary material available at 10.1007/s15010-026-02732-y.

## Introduction

Evidence shows that biological sex has an impact on the immune function. Besides playing a role in morbidity and mortality among infected patients, sex hormones also have an influence on the immune system changes during pre- and postmenopausal stages in females [1–4].

Numerous studies have suggested that females may experience favorable outcomes in comparison with males during an infection [1, 2, 5–8]. Studies in septic patients have found higher Sequential Organ Failure Assessment (SOFA) scores in post-menopausal females compared to men of the same age, which raises the question as to whether the post-menopausal stage may increase morbidity in female patients with infections [9].

Studies also found that both males of all ages and postmenopausal females (older than 50 years) were more prone to sepsis after trauma and BSI, compared to females younger than 50 years [2, 3].

A higher incidence of bloodstream infections (BSIs) and mortality following BSIs is seen among males, with a relative risk around 1.3 (95% CI 1.1–1.6, P < 0.05), compared to females. However, this increased survival among females diminishes in the post-menopausal stage [2, 8].

Understanding the sex differences in the mechanism of systemic infections may help identify areas for further research that ultimately may guide individualized therapy, by setting new biomarker thresholds or timing of treatments according to sex and age.

The theories for sex-dependent differences in inflammation, sepsis, and other models of shock (e.g., trauma-hemorrhage) largely center on the beneficial anti-inflammatory effects of the sex hormones estrogen and progesterone, as well as the potential immunosuppressive effects of testosterone [8].

Both estrogen and progesterone have shown a protective and immune-enhancing effect [10]. Animal studies have indicated that estrogen can regulate immune responses and impact a protective effect in response to infections by e.g., up-regulating anti-inflammatory cytokines such as interleukin-10 (IL-10), while reducing pro-inflammatory cytokines such as interleukin-6 (IL-6) and tumor-necrosis-factor-alph10a (TNF-α) [2, 4–6, 8, 10]. It has been suggested that progesterone, which is elevated during the luteal phase, plays a crucial role in the immune system's ability to fight infections in females. At the same time, estrogen reduces the immune response to protect the spermatozoon against the innate immune response during the ovulation phase [4, 6].

This hormonal immuno-modulating difference between men and women seems to increase until the onset of menopause in females, supporting a strong role for sex hormones [8].

Regarding inflammatory biomarkers, a study found that the average level of white blood cell count in healthy females was reduced before and up to 2 years after entering the postmenopausal stage [11].

C-reactive protein (CRP) is widely used in Denmark as an important biomarker when diagnosing, treating and tracking infections. Higher CRP levels may indicate severe infection and the need for antibiotics in suspicion of bacterial infection [12, 13].

Evidence also suggests that the sex steroid hormone estrogen can influence CRP levels, with hormone replacement therapy having a profound influence on CRP levels in the elderly, by reducing IL-6 expression, as well as TNF-α and IL-1β [4].

The literature has so far not investigated trajectory patterns of neutrophils or CRP comparing males and females with BSI.

The purpose of this study is to characterize and compare sex-specific inflammatory trajectories of CRP and neutrophils in relation to a bloodstream infection episode, and to examine whether these trajectories differ by age group as a proxy for menopausal status.

## Methods

### Setting

The Danish healthcare system is tax-based and thus free of charge for the individual patient at the point of care [14].

Denmark is divided into five regions, which administer the healthcare system of a population of approximately 6 million inhabitants. This is a population-based study based upon the fact that all patients with an acute disorder are admitted to a hospital within the region in which they reside and patients with a BSI are always admitted to a hospital. The unique personal identifier, given to all Danish residents, further enables the merging of data from health and administrative registries [15, 16].

### Study population

In this retrospective registry-based cohort study, we selected patients aged ≥ 15 years from the Region of Southern Denmark (comprising 21% of the Danish population) who had a BSI between 2007 and 2016. We only included the patients’ first BSI episode within this period. In this paper, we refer to males and females as the biological sex, and not gender in a cultural sense.

The initial study population has been described previously [17].

In brief, the core data were positive blood cultures from which we computed BSI episodes based on globally accepted algorithms [17]. These data were linked to the Danish National Patient Registry (DNPR), which has recorded all hospital admissions with their diagnoses and procedures since 1977 [20], and the Civil Registration System, with its records on the vital status as well as emigration and death dates, if relevant [15].

Finally, we linked the data to all the patients’ laboratory results for CRP and neutrophils, restricting the results to specimens retrieved from 30 days before (D–30) through 30 days after (D30) the date of sampling the positive blood culture in a BSI episode (D0).

We used the DNPR data to compute all the hospital admissions, and for the admissions with the BSI episode, we categorized the place of acquisition as community-acquired, healthcare-associated, or hospital-acquired as described previously [17]. We further retrieved the Charlson comorbidity diagnoses prior to D0 and categorized the Charlson comorbidity index into 0, 1–2, and ≥ 3 points [21].

For each patient in our study cohort, the vast majority of the two biomarkers were only measured in one specimen on a specific date within the D–30/D30 period. In the very few cases where a biomarker was measured more than once on the same date, we randomly retrieved one value.

### Statistical analyses

We initially divided our study population into females vs. males and two age groups, 15–49 and ≥ 50 years on D0, based on the assumed pre- and post-menopausal periods in females. For these four groups, we computed a contingency table for the acquisition mode, Charlson comorbidity index groups, main microorganisms, and 30 day mortality.

We categorized the microorganisms in the first-time BSI episode into eight main groups (*Escherichia coli, Staphylococcus aureus, coagulase-negative staphylococci* (CNS), *Streptococcus pneumoniae, Klebsiella spp., Enterococcus spp.*, remaining microorganisms in mono-microbial BSI episodes [“miscellaneous”], polymicrobial) and depicted their percentage distribution according to the patients’ age group in 5-year intervals, separately for females and males, in a stacked area plot.

The daily mean levels were computed for all the biomarker levels on a specific day in the D–30/D30 period.

For each of the two biomarkers, CRP and neutrophils, we graphically depicted trajectories, with and without 95% confidence intervals (CIs), based on the daily mean levels of each biomarker in the D–30/D30 period, separately for the four sex and age-generated groups. We visually assessed these trajectories to derive sub-periods with a linear course for the trajectories, as well as based on a previous study in which CRP started to increase on D–3 [22].

We computed four sub-periods based on this visual assessment: D–30/D–4, D–3/D1, D2/D5, and D6/D30. For each sub-period, we applied multilevel mixed effects linear regression analyses with the individual levels of the biomarker as the dependent variable. As fixed effects, we included sex as the main independent variable, as well as adjusting for each day in the sub-period, age on D0, the Charlson comorbidity index (0, 1–2, ≥ 3), the eight main groups of microorganisms, and the acquisition mode (community-acquired, healthcare-associated, hospital-acquired). The individual patient was the random effect. For each sub-period, we report the results as the trajectories mean daily difference (i.e., the difference of the trajectory’s slope) with its 95% CIs for males (with females as the reference group). Within each period, we defined significance if the 95% CIs of this difference did not include 0.

We used the program Stata, vs. 18 (StataCorp LLC, College Station, Texas, USA) for all the analyses.

## Results

### Baseline characteristics

Our study population included 24,074 patients who had 268,648 specimens with CRP and 138,428 with neutrophils in the D–30 to D30 day period. The lowest number of specimens was found at D–30 (1398 and 843 specimens with CRP and neutrophils, respectively), while the highest number was found at D0 (17,846 and 13,202 specimens).

There were more females in the 15–49 year age group (52.2%), whereas this was reversed in the ≥ 50 year age group (42.2%) (Table 1). In both age groups, fewer males than females had a community-acquired BSI, whereas this was reversed for hospital-acquired BSIs. Males were also more comorbid, regardless of age group. Concerning microorganisms, the main differences between the sexes were seen for *E. coli*, which was more prevalent among females, especially in the younger age group, and for *S. aureus,* which had a higher prevalence in younger males. In the 15–49 year age group, males had a higher 0–30 day mortality (8.8%) than females (5.2%), whereas there was no difference in the ≥ 50 year age group.

**Table 1:** Baseline characteristics of the study population (n = 24,074) from 2007 to 2016 stratified by sex and two age groups

	15–49 years		> 50 years	
	Females	Males	Females	Males
No. of patients (%)^1^	1397 (52.2)	1277 (47.8)	9021 (42.2)	12,379 (57.9)
Acquisition				
^Community	763 (54.6)^2^	648 (50.7)	4714 (52.3)	5616 (45.4)
^Healthcare-associated	362 (25.9)	297 (23.3)	2610 (28.9)	3740 (30.2)
^Hospital	253 (18.1)	309 (24.2)	1637 (18.2)	2924 (23.6)
^Unknown	19^ (1.4)	23 (1.8)	60 (0.7)	99 (0.8)
Charlson comorbidity index				
^0 points	703 (50.3)	521 (40.8)	1,639 (18.2)	1,964 (15.9)
^1–2 points	415 (29.7)	406 (31.8)	3,626 (40.2)	4,506 (36.4)
^≥ 3 points	277 (19.8)	350 (27.4)	3,751 (41.6)	5,905 (47.7)
Microorganisms				
^*Escherichia coli*	443 (31.7)	160 (12.5)	3189 (35.4)	3170 (25.6)
^*Enterobacter* spp.	19 (1.4)	22 (1.7)	156 (1.7)	300 (2.4)
^*Klebsiella* spp.	55 (3.9)	49 (3.8)	504 (5.6)	953 (7.7)
^Other *Enterobacterales*	47 (3.4)	49 (3.8)	270 (3.0)	646 (5.2)
^*Pseudomonas aeruginosa*	20 (1.4)	23 (1.8)	150 (1.7)	399 (3.2)
^Anaerobic Gram-negative rods	49 (3.5)	37 (2.9)	196 (2.2)	280 (2.3)
^Other Gram-negative bacteria	64 (4.6)	40 (3.1)	218 (2.4)	279 (2.3)
^*Staphylococcus aureus*	146 (10.5)	274 (21.5)	917 (10.2)	1421 (11.5)
^*Coagulase-negative staphylococci*	117 (8.4)	151 (11.8)	683 (7.6)	865 (7.0)
^*Streptococcus pneumoniae*	127 (9.1)	116 (9.1)	715 (7.9)	673 (5.4)
^*Hemolytic streptococci*	85 (6.1)	60 (4.7)	422 (4.7)	524 (4.2)
^*Enterococci*	53 (3.8)	63 (4.9)	429 (4.8)	1006 (8.1)
^Other Gram-positive cocci	40 (2.9)	51 (4.0)	229 (2.5)	401 (3.2)
^Gram-positive rods	29 (2.1)	36 (2.8)	167 (1.9)	218 (1.8)
^Fungi	26 (1.9)	33 (2.6)	188 (2.1)	293 (2.4)
^Poly-microbial	77 (5.5)	113 (8.9)	588 (6.5)	951 (7.7)
Mortality^2^				
^Day 0–30	72 (5.2)	112 (8.8)	1845 (20.5)	2,537 (20.5)

^1^Number of patients (percentage)

^2^Percentages based on denominators, which deviate from the study population due to 10 patients who emigrated before their BSI episode and 3 who emigrated 0–30, days after their BSI episode

### Microbiologic agents and age

**Fig. 1:**
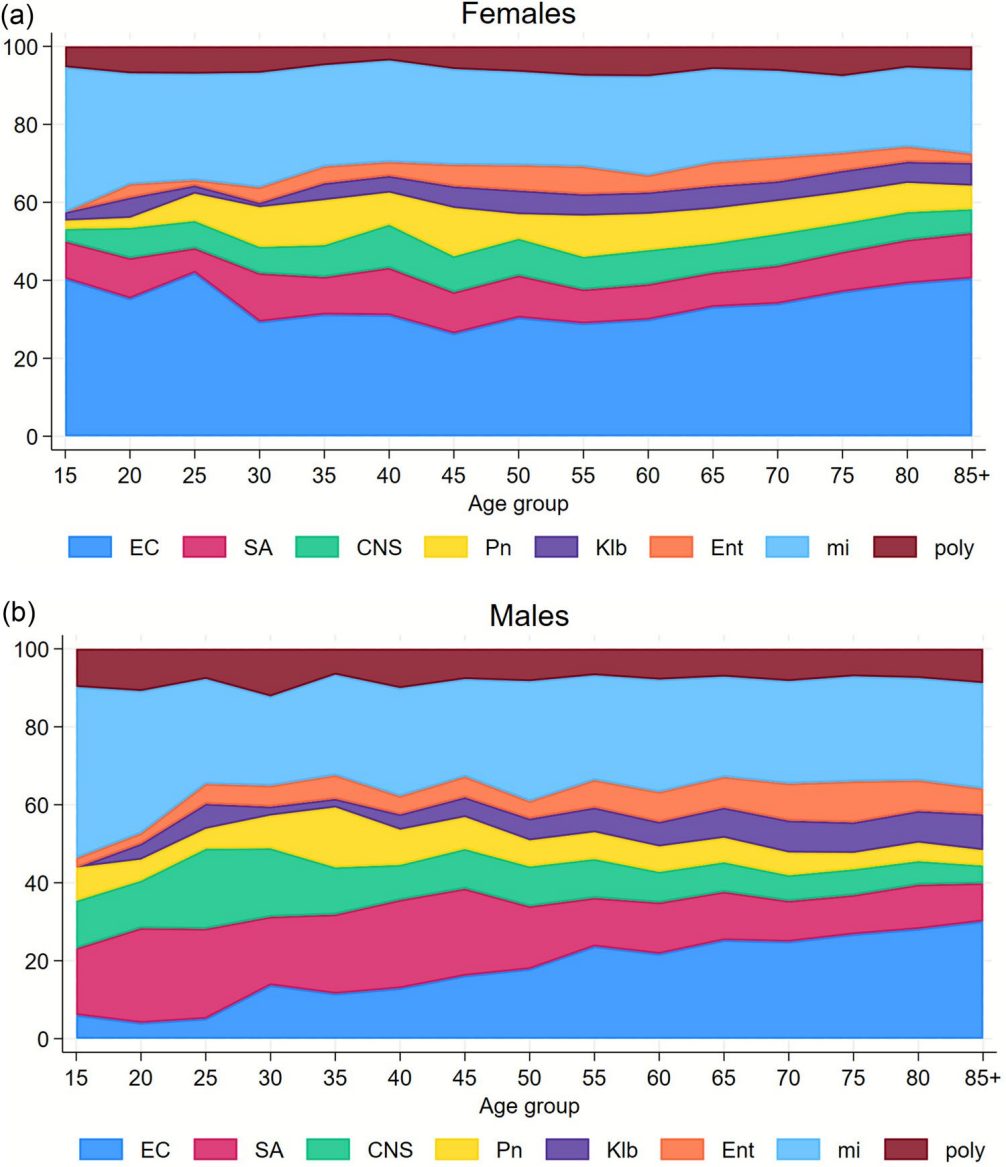
Percent distribution of main groups of microorganisms in year age groups, among females and males, aged 15 years or older. EC, *Escherichia coli*; SA, *Staphylococcus aureus*; CNS, coagulase-negative staphylococci; Pn, *Streptococcus pneumoniae*; Klb, *Klebsiella* spp.; Ent, *Enterococcus* spp.; mi, Miscellaneous; poly, polymicrobial

Distribution of microbiological agents in relation to age varied in our population groups as follows (Fig. 1): *E. coli* had a higher prevalence in females compared to males throughout all ages, though increasing throughout age in males. Among patients aged up to 50 years, *S. aureus* and CNS were more prevalent in males than in females, whereas this difference minimized thereafter. The distributions of the other microorganisms, *S. Pneumoniae, Klebsiella spp***.,** *Enterococcus spp*., miscellaneous, and polymicrobial, were more or less equal.

### CRP trajectories in the 15–49 year age group, descriptive part

**Fig. 2:**
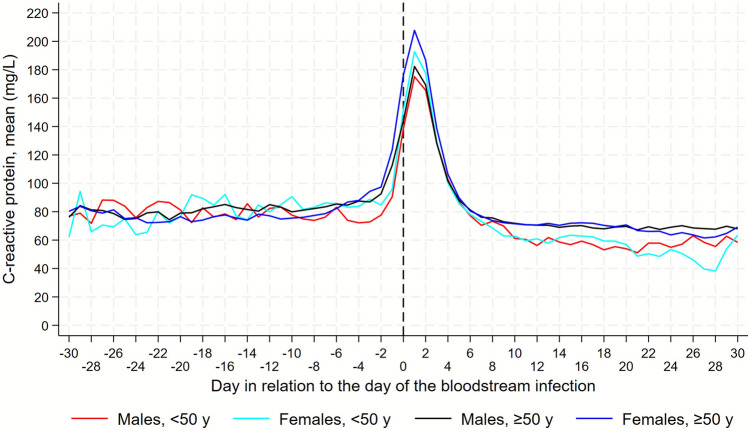
Daily mean levels of C-reactive protein among males and females. Separate trajectories for females and males, under and over 50 years. Day 0 is the date of sampling of the first positive BC in a BSI episode. Trajectory starts at day–30 and continues until day + 30

The daily mean CRP values did not differ considerably between the sexes in the D–30/D–4 period (Fig. 2, Fig. 1S).

In the period D–3/D1, the biggest differences were observed. For both males and females, the CRP level began to increase at D–2, males starting from a lower initial level (77.6 mg/L) than females (84.8 mg/L). The females exceeded males of the same age group with 17.6 mg/L (10.9%).

At D1, CRP levels peaked for both sexes with females exhibiting the highest level, 192.7 mg/L, vs. 175.1 mg/L for males. Beyond D2, females and males had similar trajectories and CRP levels.

### CRP trajectories in the ≥ 50-year age group, descriptive part

Before D–3, the trajectories and levels were similar between the two sexes (Fig. 2, Fig. 1S).

The increase from D–3 through D1 was higher in females than in males, reaching a peak level of 207.7 mg/L and 182.3 mg/L, respectively, by this, the females exceeded peak level of males with 25.4 mg/L (8,5%).

In the D2/D5 period, females still had somewhat higher CRP levels, after which there were no considerable differences between the two sexes.

### Neutrophil trajectories in the 15–49 year age group, descriptive part

**Fig. 3:**
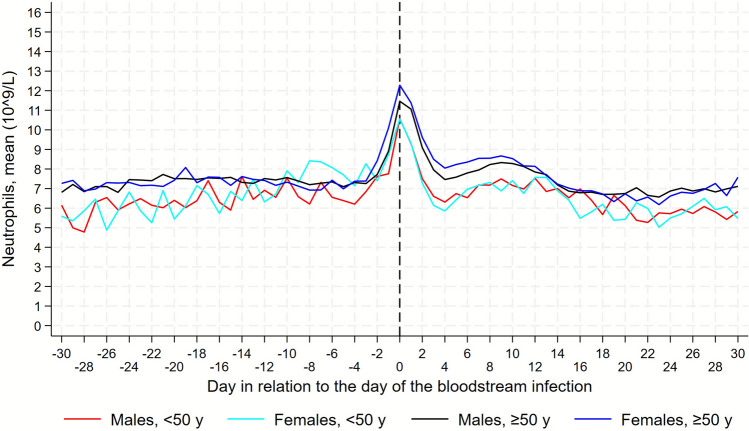
Daily mean levels of neutrophil trajectories among males and females. Separate trajectories for females and males, under and over 50 years. Day 0, the date of sampling the positive blood culture in a BSI episode. Trajectory starts at day–30 and continues until day + 30

From D–30/D–10, females tended to have slightly lower neutrophil levels compared to males (Fig. 3, Fig. 2S). From D–10, neutrophil levels in females slowly began to increase, whereas males exhibited a steeper increase, starting at D–4.

In the period D–3/D1, both males and females peaked at D0, both with 10.6 × 10^9/L, followed by a similar decrease thereafter.

From D4/D12 among males and D4/D13 among females, the neutrophil levels briefly increased in both sex groups, before gradually decreasing throughout the period until D30.

### Neutrophil trajectories in the ≥ 50 year age group, descriptive part

During the first period, D–30/D–4, females exhibited lower levels until D–11, although the differences between the sexes were smaller than compared to the group 15–49 years (Fig. 3, Fig. 2S).

At D–10, neutrophils of males began a slow increase whereas neutrophils of females first began to increase around D–4/D–3.

In the period D–3/D1, neutrophil levels peaked for both sexes at D0, with females exhibiting a higher neutrophil level than males, 12.3 × 10^9/L and 11.5 × 10^9/L, respectively.

From D2/D5 neutrophil levels decreased in both sexes, reaching the lowest level at D4, at 8.5 × 10^9/L for males and 8.0 × 10^9/L for females. From D4/D10, the neutrophil levels briefly increased in both sex groups, before gradually decreasing throughout the period until D30.

### Results from the multilevel mixed-effects linear regression

The results from these analyses, in which we adjusted for possible confounders, generally confirmed the ones shown in Figs. 2, 1S, 3, and 2S (Table 2).

**Table 2:** Multilevel mixed-effects linear regression analyses for daily mean levels of CRP and neutrophils from 30 days before through 30 days after the patients’ first-time bloodstream infection

Time span	C-reactive protein (mg/L)				Neutrophils (10^9/L)		
	N1/N2^1^	Slope^2^ mg/L/day (95% CI)^2^				N1/N2	Slope^2^ 10^9/L/day (95% CI)
*Patients aged < 50 years*							
Day–30/–4	6161/1026	3.2 (− 5.8/12.2)				3222/869	0.55 (− 0.08/1.18)
Day–3/1	5068/2071	**9.6 (0.61/18.6)**				3019/1683	− 0.14 (− 0.80/0.52)
Day 2/5	5701/1909	3.0 (− 4.6/10.7)				2820/1170	− 0.18 (− 0.79/0.43)
Day 6/30	11,744/1648	− 2.5 (− 7.9/2.8)				6156/1278	0.07 (− 0.42/0.56)
*Patients aged ≥ 50 years*							
Day–30/–4	50,406/5240	0.3 (− 2.4/3.1)				27,495/7874	0.13 (− 0.11/0.36)
Day–3/1	40,494/16,798	**23.8 (20.6/27.1)**				24,268/13,692	**0.49 (0.25/0.73)**
Day 2/5	47,285/15,455	**9.3 (6.7/11.9)**				22,383/9290	**0.50 (0.26/0.74)**
Day 6/30	100,592/13,721	0.1 (− 1.8/2.0)				48,312/10,206	**0.22 (0.02/0.41)**

^1^Number of specimens/number of patients

^2^Slope (95% confidence interval) for females (reference group: males), in models adjusted for days in the time span (the table’s first column), age, comorbidity, main groups of microorganisms, and place of acquisition. Results in bold are significant

Among the age group < 50 years, a significantly higher mean daily difference of 9.6 mg/L/day for CRP was seen among females, in comparison to males during the period D–3/D1.

Further, significant mean daily differences were seen among the age group ≥ 50 years, both regarding CRP and neutrophils. The CRP mean daily difference during the D–3/D1 period was 23.8 mg/L higher in females than males, which continued in the next period (D2/D5) with 9.3 mg/L more in females.

Regarding neutrophils, also in the age group ≥ 50 years, females exhibited a slightly higher mean daily difference during the periods D–3/D1 and D2/D5 at 0.49 × 10^9/L and 0.50 × 10^9/L, respectively. During the last period (D6/D30), the mean daily difference of females declined, but it still exceeded the one for males with 0.22 × 10^9/L.

## Discussion

### Main findings

In this population-based study of BSI and sex and age specific differences in the CRP/neutrophil trajectories, we found that. females ≥ 50 years exhibited a faster CRP response, and a higher peak level compared to both males of both age groups and females < 50 years. Females of both age groups exhibited higher CRP peak levels at D1, compared to males of the same age. Females ≥ 50 years had the highest mean peak level of all four groups. When it comes to neutrophils, females ≥ 50 years also had the highest peak level at D1. When looking at the CRP trajectories and response patterns, females and males ≥ 50 exhibited their initial/earliest CRP increase 1 day earlier (D–3) than their corresponding younger groups (D–2). Though when it comes to neutrophils, females < 50 exhibited their earliest response in neutrophil increase, compared to males of the same age group and the ≥ 50 females.

Given the higher inflammatory peaks in females and males > 50, inflammaging and immunosenescence might have an amplifying effect on the inflammatory response.

Furthermore, there may be non-hormonal explanations for differences in the levels of CRP or neutrophils. As examples of non-hormonal factors, we accounted for differences in distribution of microorganisms and acquisition mode in our multivariate analyses, but unknown confounders, e.g. infectious foci, may also have an impact.

Our findings overall may disprove our hypothesis that hormones of pre-menopausal have an immune effect regarding infections. Of note, mortality rates within the first 30 days were lower for females < 50 years, compared to males in the same age group. Our study did not elucidate prognostic aspects, but further studies that link inflammatory markers to prognosis in relation to sex and age groups are warranted.

### Biological sex differences in immune function during sepsis

Female sex hormones regulate the immune system, e.g. by differences in the lifespan of neutrophils, exhibiting delayed neutrophil apoptosis in females, when compared to males [4, 6].

Unfortunately, the picture at the bedside is more complex. Numerous studies have found a favorable outcome in terms of dealing with infections among females compared to males [1, 2, 5–7]. although a few studies found no biological sex-derived clinical or biochemical differences in inflammatory response among septic patients and some even found a worse outcome among females [2, 4, 6].

This may suggest that sex hormones act differently, depending on the context and moreover confounded by other variables, such as the causative microorganism, infection focus, age, acquisition, treatment, or time in menstrual cycle for fertile females.

### CRP and neutrophil differences in general

To our knowledge, only one study has assessed CRP levels in males vs. females with BSI and only for *S. aureus* on D0 [23]. Smit et al. [23] found that females exhibited a median CRP level of 184.0 mg/L on D0, in contrast to 170.3 for males, which corroborates our findings. Of note, that was also a Danish study, with a setting similar to ours.

To our knowledge, there is no currently available scientific literature, which has systematically tracked and compared neutrophil dynamics over time between males and females. This represents an important area for further investigations, as sex-based differences in immune function could potentially influence clinical outcomes, treatment responses, or the development of personalized therapeutic approaches in BSI management.

### Microbiologic differences

Surprisingly we found a decrease in *S*. *aureus* in males > 50 compared to the younger ones. We would expect it to increase from a clinical view, as older males are in risk of more infections related to e.g. catheterizations. One explanation might be that a higher proportion among the younger males with BSI might be caused by trauma, and thereby possible skin related BSI. Unfortunately we don’t have clinical data to prove or refute this fact,

The increase in *E. coli* in older men compared to the younger, might be explained by increased urinary tract deficits related to physiologic growth of the prostate and thereby increased residual urine, causing good conditions for colonization of *E*. *coli*.

## Strengths of our study

Our study was population-based, with a high number of patients and data recorded independently from the study questions.

Further, the linkage to the Civil Registration System with its data on vital status enabled follow-up even after discharge from the hospital. Moreover, BSI represents a serious infection unanimously diagnosed and defined according to generally accepted microbiological criteria[17–19] in contrast to sepsis criteria based on clinical criteria of “organ dysfunction” that are vividly debated [24].

## Limitations of our study

Firstly, there is a lack of data on the exact reproductive stage and stage in the menstrual cycle for females. We set 50 years as the assumed point of division between peri- and postmenopausal stages serving as proxies.

Secondly, we lacked clinical data, which is a general limitation of data based on administrative registries. As an example, our data did not include sex hormones, cytokines, or related biochemical mediators and consequently our study should be interpreted as hypothesis-generating. Other study types, such as prospective studies, are needed to confirm or refute the suggested hypotheses.

Thirdly, confounding by indication is a caveat when numbers of and time intervals between biomarker specimens in these real-life data are closely related to the severity of the underlying patient’s illness. From that perspective, data were not missing at random [25]. However, the main issue of the present study is comparisons between days and time periods in relation to a BSI and in this context there is no reason to believe that the clinician’s threshold for retrieving a biomarker specimen is associated to a specific day within the D−30/D30 period. A previous study, in which missing data had little impact on trajectory patterns, corroborated this assumption [26]. Moreover, the differences were seen on the days with the highest numbers of blood specimens, i.e. with the lowest thresholds for their retrieval, which also mitigates this caveat.

Fourthly, in spite of the population-based design, biomarker specimens were not retrieved within D–30/D30 of the first BSI episode from 4395 BSI patients in the study period (data not shown) and our study population is therefore likely to comprise the frailer patients.

Fifthly, the number of specimens retrieved before the BSI episode differed in relation to variables seen in Table 1, e.g., the acquisition mode (community-acquired, healthcare-associated, and hospital-acquired). We cannot evaluate the impact of this on the trajectories, but CIs were reasonably narrow in the pre-BSI period for all subgroups.

Finally, trajectories and analyses comprising the post-BSI period should be interpreted with even more caution than the pre-BSI results, as many patients died or were discharged alive within the 30 days, resulting in a continuously changing study population.

## Conclusion

Females in general exhibited slightly higher CRP levels, than males, during BSI. Regarding neutrophils, the highest peak was not correlated to sex, but rather to the eldest age groups, compared to the < 50 years age group, while the younger group, < 50, of both sexes exhibited a faster peak response.

Our findings support the importance of stratifying patients into sex and age groups based on pre- and post-menopause in females when evaluating inflammatory responses to an infection. Further studies that include sex hormones, cytokines and related biochemical mediators are needed to gain a better comprehensive physiological understanding of the sexual differences in the inflammatory response during BSI. This may identify target therapies, which could supplement the existing antibiotic treatment. Of note, our study is hypothesis-generating and prospective studies of patients with BSI with the inclusion of various clinical variables, e.g., sepsis, are needed to confirm or refute our findings.

## Data availability statement

The data that support the findings of this study are not openly available due to reasons of sensitivity and are available from the corresponding author upon reasonable request. Data are located in controlled-access data storage at Statistics Denmark.

## Supplementary Information

Below is the link to the electronic supplementary material.

Supplementary file1 (DOCX 27369 KB)

## Declarations

### CRediT taxonomy

Conceptualization: [Cathrine Sandager Budtz, Line Riis Jølving, and Kim Oren Gradel]; Methodology and formal analysis: [Kim Oren Gradel]; Writing -original draft preparation: [Cathrine Sandager Budtz]; Writing -review and editing: [Line Riis Jølving, Kim Oren Gradel, Pedro Povóa, Ram Benny Christian Dessau, John Eugenio Coia, Jens Kjølseth Møller, Stig Lønberg Nielsen]; Resources: [Kim Oren Gradel and Line Riis Jølving]. Supervision: [Kim Oren Gradel and Line Riis Jølving].
