# Supplementary material for: Sex and age differences of inflammatory biomarkers around a bloodstream infection: a population-based cohort study
Source: Infection. 2026 Jan 20;54(2):941–50. doi: 10.1007/s15010-026-02732-y (PMC13021796; doi:10.1007/s15010-026-02732-y)
Supplement: Supplementary file 1 — Supplementary file1 (DOCX 27369 KB) [file 15010_2026_2732_MOESM1_ESM.docx]

**Sex and age differences of inflammatory biomarkers around a bloodstream infection – a population-based cohort study**

**Original paper 2025 Infection**  **-a journal of infectious diseases**

Cathrine Sandager Budtz^1,10^, Line Riis Jølving^1,2^, Pedro Póvoa^1,3,4^, Stig Lønberg Nielsen^5,6^, Ram Benny Dessau^7, 11^, Jens Kjølseth Møller^8,9^, John Eugenio Coia^9^, Kim Oren Gradel^1,2^

**Author affiliation**

^1^Center for Clinical Epidemiology, Odense University Hospital, ^2^Research Unit of Clinical. Epidemiology, Department of Clinical Research, University of Southern Denmark, Odense C, Denmark, ^3^The Polyvalent Intensive Care Unit, Hospital de São Francisco Xavier, CHLO, Estrada do Forte do Alto do Duque, Lisbon, Portugal , ^4^NOVA Medical School, New University of Lisbon, Lisbon, Portugal, ^5^Department of Infectious Diseases, Odense University Hospital, ^6^Research Unit of Infectious Diseases, Department of Clinical Research, University of Southern Denmark, Odense, Denmark, ^7^Department of Clinical Microbiology, Zealand University Hospital, Slagelse, Denmark ,^8^ Department of Clinical Microbiology, Vejle Hospital, University Hospital of Southern Denmark, Vejle, Denmark, ^9^Institute for Regional Health Research, University of Southern Denmark, Odense, Denmark, ^10^Department of Radiology, University Hospital of Southern Denmark, Kolding, Denmark, ^11^Department of Regional Health Research, University of Southern Denmark

**Corresponding author:**

Kim Oren Gradel

Clinical epidemiologist, associate professor, senior DVM, Ph.D.

Center for Clinical Epidemiology and Research Unit of Clinical epidemiology

OUH Odense University Hospital

Kløvervænget 30, Entrance 216, ground floor,

DK-5000 Odense C

Email: Kim.gradel@rsyd.dk Kim.gradel@rsyd.dk

Phone: +45 21 15 80 85

**Supplementary**

**Figure 1 S**  Daily mean levels of CRP trajectories incl. confidence intervals, among males and females. Separate trajectories for females and males, under and over 50 years. Day 0, the date of sampling the positive blood culture in a BSI episode. Trajectory starts at day – 30 and continues until day +30.

**
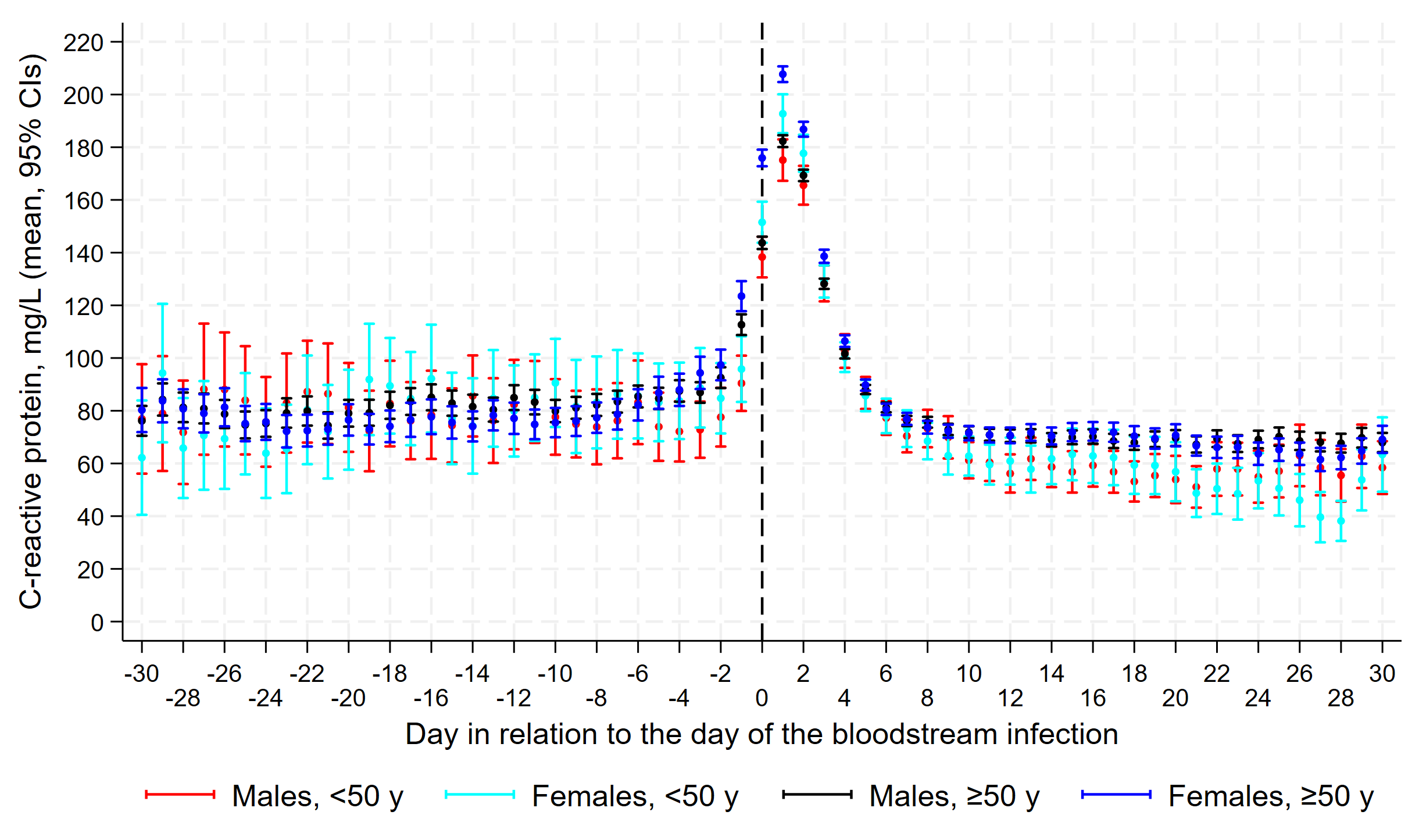
**

**Figure 2 S** Daily mean levels of neutrophil trajectories incl. confidence intervals, among males and females. Separate trajectories for females and males, under and over 50 years. Day 0, the date of sampling the positive blood culture in a BSI episode. Trajectory starts at day – 30 and continues until day +30.

**
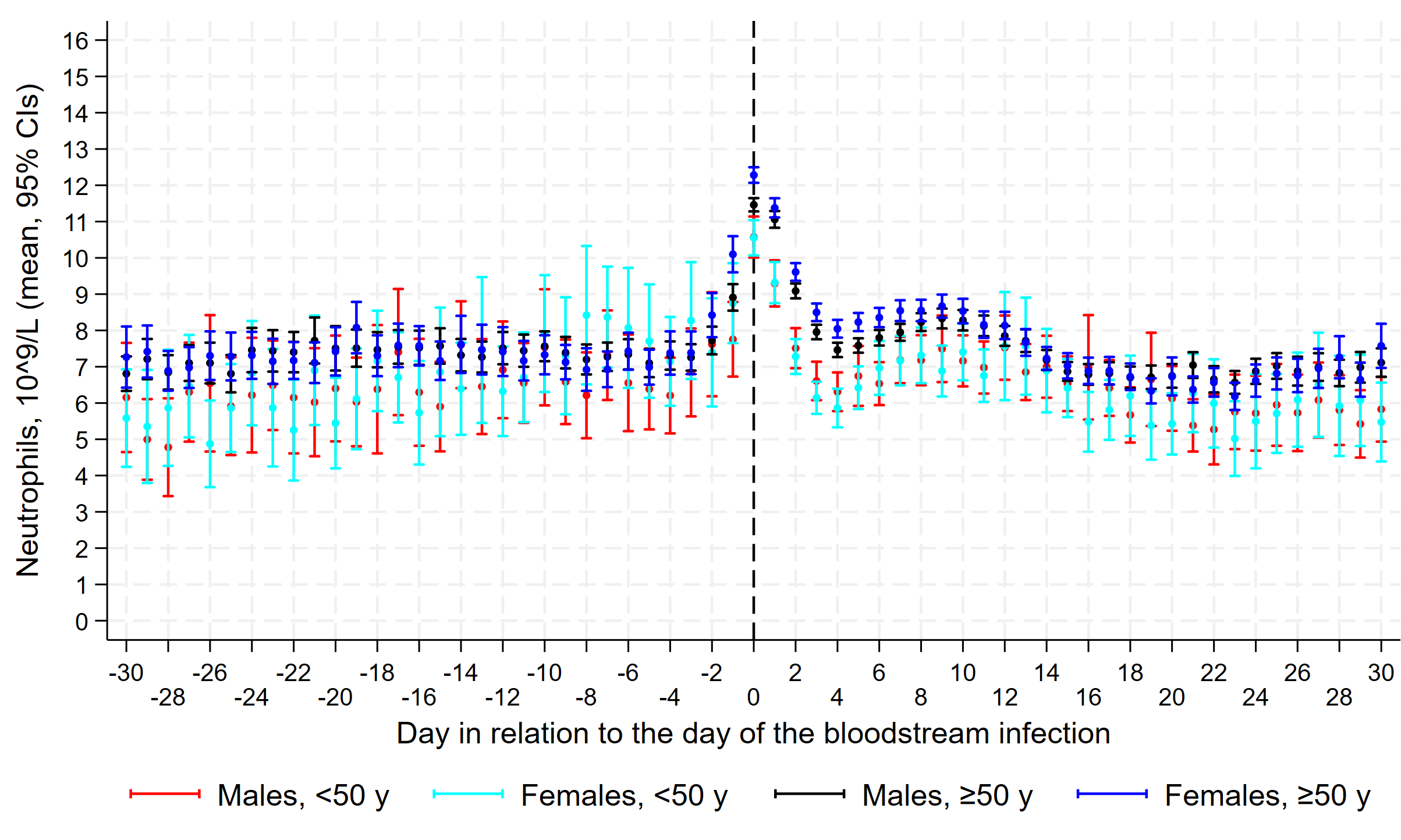
**
